# Minipuberty in Daughters of Women with Hypothyroidism during Pregnancy

**DOI:** 10.3390/ijms25158244

**Published:** 2024-07-28

**Authors:** Karolina Kowalcze, Robert Krysiak, Joanna Kula-Gradzik, Giuseppe Gullo

**Affiliations:** 1 Department of Pediatrics in Bytom, Faculty of Health Sciences in Katowice, Medical University of Silesia, Stefana Batorego 15, 41-902 Bytom, Poland; jkula-gradzik@sum.edu.pl; 2 Department of Pathophysiology, Faculty of Medicine, Academy of Silesia, Rolna 43, 40-555 Katowice, Poland; 3 Department of Internal Medicine and Clinical Pharmacology, Medical University of Silesia, Medyków 18, 40-752 Katowice, Poland; rkrysiak@sum.edu.pl; 4 Department of Obstetrics and Gynecology, Villa Sofia Cervello Hospital, University of Palermo, 90146 Palermo, Italy

**Keywords:** genital organs, hypothalamic–pituitary–ovarian axis, girls, hypothyroidism, pregnancy complications, saliva

## Abstract

Minipuberty is a term describing transient postnatal activation of the hypothalamic–pituitary–gonadal axis, likely playing an important role in the postnatal growth of female genital organs and breasts. Unlike infant boys, there are no data concerning the impact of gestational hypothyroidism on the course of minipuberty in infant girls. Therefore, the aim of the current study was to investigate the reproductive axis and genital organs in daughters of women with thyroid hypofunction during pregnancy. The study population included three matched groups of infant girls: offspring of women with thyroid hypofunction non-substituted or inadequately treated during gestation (group 1), descendants of women adequately substituted throughout pregnancy (group 2), and daughters of healthy women (group 3). Salivary concentrations of estradiol, progesterone, 17-hydroxyprogesterone, and androgens (testosterone, androstenedione, and dehydroepiandrosterone sulfate) and urine levels of gonadotropins were measured monthly from month 1 to month 6, once every two months between postnatal months 6 and 12, and once every three months between postnatal months 12 and 18. During each visit, we also determined ovarian volume, uterine length, and breast diameter. Concentrations of FSH, LH, and estradiol were lowest in group 1, and this group was also characterized by the shortest detection period for gonadotropins and estradiol. These differences were paralleled by differences in ovarian volume, uterine length, and breast diameter. There were no differences between groups 2 and 3 in levels of both hormones and in the size of the measured structures. The obtained results seem to indicate that non-substituted or inadequately substituted hypothyroidism during pregnancy may impair the course of minipuberty in the female offspring.

## 1. Introduction

Minipuberty of infancy is a transient sex-specific phase of postnatal activation of the reproductive axis. Concentrations of both pituitary gonadotropins, follicle-stimulating hormone (FSH) and luteinizing hormone (LH), start to rise in the first few days after birth in response to disappearance of the suppressive effect of placental hormones, resulting in stimulation of the gonads [1,2,3,4]. Peak concentrations of FSH in infant girls in this period of life are higher than those of LH, while the subsequent increase in estradiol levels reaches values observed during Tanner stage 4 [3,4]. FSH and estradiol levels remain elevated until the ages of 3–4 and 2 years, respectively, while LH decreases to prepubertal levels at around the age of 6 months [3]. After completing minipuberty, the hypothalamic–pituitary–gonadal axis activity remains in a state of relative quiescence until reactivation at puberty [1]. The biological significance of minipuberty in girls is still insufficiently understood. It seems to play an important role in the postnatal development of the uterus and breasts [5]. Hormonal changes associated with minipuberty may also determine sex differences in growth velocity and the pattern of body adipose tissue distribution [2]. Lastly, minipuberty probably plays a role in forming brain plasticity and in the development of cognitive abilities and emotional competences [4].

No previous study has investigated a relationship between exposure to subnormal levels of thyroid hormones and the course of minipuberty in girls. However, the results of a nationwide Danish cohort study showed no differences in pubertal development between daughters born to women with thyroid disorders (including hypothyroidism) and female descendants of healthy women [6]. Unfortunately, the data were not collected by the researchers (the study was register-based), which might have affected their quality. Moreover, the study design made it difficult to differentiate between prevalent and incident cases [7]. Consequently, some women might have been misdiagnosed with hypothyroidism, and the population of hypothyroid women probably differed in substitution effectiveness. In turn, animal studies assessing genital maturation of female offspring of dams with gestational hypothyroidism provided inconsistent results. Some authors observed differences in the volume, size and weight of ovaries, as well as in the number of secondary follicles between the offspring of females with propylthiouracil-induced gestational hypothyroidism and those of healthy females [8,9]. Furthermore, the diameter of Graafian follicles in young female rodents was greater if dams were exposed in utero to propylthiouracil [10]. However, timing of vaginal opening, a widely used external sign of puberty, was not affected by prenatal expose to propylthiouracil-induced maternal hypothyroidism [11]. Lastly, female offspring of animals receiving propylthiouracil and melatonin during pregnancy reached puberty, became anestrus, and returned to cyclicity at similar times to contemporary ewe lambs [12].

Some clinical observations may indirectly suggest an association between gestational hypothyroidism and the course of female minipuberty. Thyroid hypofunction in pregnant women is associated with an increased risk of premature delivery and abnormal (usually high) birth weight [13,14]. In turn, premature female infants are characterized by higher gonadotropin and estradiol concentrations than full-term ones [15,16], which is suggestive of increased activity of the hypothalamic–pituitary–gonadal axis at the hypothalamic or pituitary level. Moreover, although there are no data for children with high birth weight, infant girls born small for gestational age had higher FSH and sex hormone levels than those born appropriate for gestational age [17]. Lastly, decreased growth velocity and impaired neurocognitive development are typical features of untreated or undertreated congenital hypothyroidism [18]. 

The lack of dedicated human studies and the inconsistent results of animal ones encouraged us to investigate whether maternal hypothyroidism during pregnancy impacts the course of minipuberty in their female offspring. The current study, including only girls, is a parallel study to our recently published work that showed that gestational thyroid hypofunction affected the activity of the reproductive axis and the development of genital organs in infant boys [19].

## 2. Results

Four children (two from group 1, one from group 2, and one from group 3) dropped out because they did not participate in the required number of visits. Two children (assigned to group 2 and 3) were withdrawn because they developed asthma and gastroesophageal reflux, requiring chronic pharmacotherapy. One child (from group 1) was withdrawn because 6 months after birth, her mother was diagnosed with Rathke cleft cyst, which was probably present earlier and might have affected the hypothalamic–pituitary–thyroid axis activity during pregnancy. The last child (assigned to group 2) prematurely terminated the study because of going abroad. The results of the remaining 88 infant girls (91.6%) were statistically analyzed.

There were no differences between the study groups in gestational age of delivery, birth order, anthropometric measures (length, weight, weight for length percentile and head circumference), whole-blood thyrotropin concentration between days 3 and 5 after birth, and in the frequency of breastfeeding (Table 1).

The study groups were comparable with respect to maternal age, education, employment, smoking, body mass index (BMI), and blood pressure (Table 2). Groups 1 and 2 did not differ statistically in conditions underlying thyroid hypofunction. Thyroid autoimmunity was reported in 15 women (52%) in group 1 and in 14 women (48%) in group 2, partial or total thyroid removal in 9 women (31%) in group 1 and in 8 (28%) women in group 2, and thyroid aplasia/hypoplasia in 3 women (10%) in group 1 and in 4 women (14%) in group 2, whereas genetic defects in the synthesis of thyroid hormones in 2 women (7%) in group 1 and in 3 women (10%) in group 2.

Estradiol was detectable in saliva during the first six months of life in group 1 and during the first 10 months in groups 2 and 3. Stable levels were observed from month 1 to month 4 in group 1 and from month 1 to month 5 in the remaining groups, and decreased thereafter. From month 1 to month 10, estradiol concentrations were lower in group 1 than in groups 2 and 3 (Table 3).

The remaining steroid hormones were detectable in saliva from month 1 to month 5 (testosterone and androstenedione) and from month 1 to month 12 (dehydroepiandrosterone sulfate [DHEA-S], progesterone, and 17-hydroxyprogesterone). Their concentrations and the detection periods were similar in all study groups and remained stable over the entire detection period (Table 4, Table 5, Table 6, Table 7 and Table 8).

FSH was detectable in urine for the first 10 months of life in group 1 and for the first 12 months of life in groups 2 and 3. Stable hormone concentrations were observed from month 1 to month 8 in group 1 and from month 1 to month 10 in the remaining groups. During the first 12 months of life, FSH concentrations were lower in group 1 than groups 2 and 3 (Table 9). 

Urinary LH levels were detectable from month 1 to month 5 in group 1 and from month 1 to month 8 in groups 2 and 3. In all study groups, LH concentrations were stable between month 1 and month 5 and decreased thereafter. During the whole period of detection, urinary LH concentrations were lower in group 1 than in the remaining two groups (Table 10).

In group 1, ovarian volume remained stable for the first 6 months and decreased thereafter. In groups 2 and 3, ovarian volume was greater between month 2 and month 10 than in months 1, 12, 15, and 18. Between months 2 and 18, ovarian volume was smaller in group 1 than in the remaining groups. There were no differences in ovarian volume between groups 2 and 3 (Table 11).

There was a decrease in uterine length between month 1 and month 5 in group 1 and between month 1 and month 2 in the remaining groups, with no changes in uterine length thereafter. From month 5 to month 18, uterine length was smaller in group 1 than in the remaining groups. There were no differences in uterine length between groups 2 and 3 (Table 12).

In all study groups, there was a decrease in breast diameter between month 1 and month 2, with no changes in this diameter thereafter. From month 5 to month 18, breast size was smaller in group 1 than in the remaining groups. There were no differences in breast diameter between groups 2 and 3 (Table 13).

There were no differences in concentrations of estradiol and gonadotropins between daughters of women with hypothyroidism untreated or inadequately treated during pregnancy resulting from autoimmune thyroid disease (n = 15) and of non-autoimmune origin (Table 14).

Over the entire detection period, salivary estradiol concentrations positively correlated with urinary LH concentrations (group 1: r values between 0.46 [*p* = 0.0001] and 0.62 [*p* < 0.0001] depending on time point [dotp]; group 2: r values between 0.50 [*p* < 0.0001] and 0.67 [*p* < 0.0001] dotp; group 3: r values between 0.51 [*p* < 0.0001] and 0.69 [*p* < 0.0001] dotp). There were also positive correlations between ovarian volume and concentrations of LH (group 1: r values between 0.29 [*p* = 0.0424] and 0.43 [*p* = 0.0008] dotp; group 2: r values between 0.30 [*p* = 0.0402] and 0.40 [*p* = 0.0011] dotp; group 3: r values between 0.32 [*p* = 0.0346] and 0.46 [*p* = 0.0002] dotp) and FH (group 1: r values between 0.27 [*p* = 0.0498] and 0.39 [*p* = 0.0015] dotp; group 2: r values between 0.28 [*p* = 0.0464] and 0.38 [*p* = 0.0014] dotp; group 3: r values between 0.28 [*p* = 0.0471] and 0.40 [*p* = 0.0012] dotp). Lastly, there were positive correlations between uterine length and estradiol concentration (group 1: r values between 0.28 [*p* = 0.0424] and 0.41 [*p* = 0.0008] dotp; group 2: r values between 0.36 [*p* = 0.0242] and 0.46 [*p* = 0.0003] dotp; group 3: r values between 0.37 [*p* = 0.0024] and 0.49 [*p* = 0.0001] dotp), between breast diameter and estradiol concentration (group 1: r values between 0.26 [*p* = 0.0495] and 0.38 [*p* = 0.0228] dotp; group 2: r values between 0.38 [*p* = 0.0023] and 0.48 [*p* = 0.0001] dotp; group 3: r values between 0.38 [*p* = 0.0027] and 0.50 [*p* < 0.0001] dotp), and in two groups between breast diameter and FSH concentration (group 2: r values between 0.31 [*p* = 0.0286] and 0.44 [*p* = 0.0006] dotp; group 3: r values between 0.29 [*p* = 0.0426] and 0.40 [*p* = 0.0014] dotp). The remaining correlations were insignificant.

## 3. Discussion

In the current study, we observed remarkable differences in the detection period for salivary steroid levels in the infant girls. The detection period was longest (12 months) for DHEA-S, progesterone, and 17-hydroxyprogesterone. This finding is in line with the results of our recent studies including boys, in whom these steroids were detectable at all time points, but the study lasted only 12 months [19,20]. In turn, detectable concentrations of the remaining steroids in daughters of healthy mothers were found only during the first ten (estradiol) or only during the first five (testosterone and androstenedione) months of life. Interestingly, estradiol levels were higher, while testosterone much lower than salivary concentrations of these hormones in peer boys [20]. These findings are probably clinically relevant. Unconjugated and protein-unbound steroid hormones easily cross blood capillaries and epithelial cells of salivary acini, and therefore their salivary content reflects the biologically active plasma pool of these hormones [21,22]. Among the assessed steroids, only DHEA-S is lipid-insoluble, owing to esterification [21]. Although its concentration in saliva is over 100 times lower than that of unbound DHEA-S in plasma [21], relatively high levels in the participants of the current study may be explained by much higher production in comparison with other steroid hormones, and possibly also by paracellular transport through tight junctions. Based on measurements in saliva, some conclusions may be drawn. Firstly, the salivary steroid profile in infancy changes with age, is specific for each steroid hormone, and differs between girls and boys. Thus, reference values for these hormones in the first 12 months of life should consider age and sex. Secondly, undetectable amounts of salivary estradiol, testosterone, and androstenedione in older infants do not have to mean their complete absence. It is probable that these steroid hormones would be detectable in assays with lower cut-off values than in those used in the current study. Lastly, abundant salivation in the first months of life, as well as easy and painless collection, indicates that saliva may be a material of choice for assessment of endogenous steroid production in infancy.

Although some peptides, particularly insulin, are actively transferred from the place of production into the salivary glands and secreted by exocytosis, this phenomenon has not been reported in the case of gonadotropins [23,24]. Their sequestration in the salivary glands is delayed in comparison with steroid hormones, and this lag time period may have an impact on the detection of relationships between gonadotropins and other assessed biomarkers [21]. Thus, gonadotropin levels were assessed in urine, the collection of which is painless and does not generate stress associated with multiple blood collections. Moreover, compared to serum, urine measurements are less subject to variability resulting from pulsatile gonadotropin (mainly FSH) secretion, which is characteristic for normal female minipuberty [2]. In line with previous observations [25], we have observed differences in the detection period of both gonadotropins, which was longer for FSH than LH (12 vs. 8 months in case of the offspring of healthy women and 10 vs. 5 months if maternal hypothyroidism was uncontrolled or poorly controlled). The presence of correlations between estradiol in morning saliva and LH in morning urine in all study groups supports the accuracy of the applied methodology.

The major finding of the current study is, however, that untreated or inadequately substituted maternal hypothyroidism affects the course of female minipuberty. Daughters of mothers with thyrotropin levels repeatedly exceeding the upper reference limit for pregnant women recommended by the American Thyroid Association [26] had lower concentrations of FSH, LH, and estradiol compared with daughters of healthy women. Moreover, gonadotropins and estradiol were detectable for a shorter period of time in the descendants of women with impaired than with normal thyroid function during pregnancy. Interestingly, most mothers with untreated or undertreated thyroid hypofunction were characterized by only mildly increased thyrotropin levels and met the criteria of subclinical hypothyroidism. Thus, it seems that, during pregnancy, all, even mild, cases of hypothyroidism should be detected and adequately managed. Interestingly, impaired activity of the hypothalamic–pituitary–gonadal axis cannot be explained by early postnatal changes in thyroid function because the study groups did not differ in whole-blood thyrotropin levels obtained between days 3 and 5 of life. The obtained results are generally in line with our previous observations concerning sons of hypothyroid mothers [19]. However, infant girls may be affected to an even greater degree than infant boys because in addition to differences in hormone concentrations, they were characterized by earlier completion of minipuberty (Table 15). During the first half of pregnancy, the fetus cannot yet produce sufficient amounts of thyroxine and triiodothyronine and is reliant on maternal thyroid hormones [26]. Thus, decreased thyroid hormone availability may disturb the initial phase of reproductive axis activation in the early and mid-pregnancy, preceding the second phase (minipuberty) and possible modulating its course [4,27]. 

In line with previous observations concerning serum and urine [16,28,29], the current study has shown detectable saliva estradiol concentrations that are higher and present for a longer period of time in daughters of women with normal thyroid function than in the offspring of women with thyroid hypofunction. This finding contrasted with no between-group differences in salivary androgens. However, it is in line with detection of analogous differences in ovarian volume, correlating with gonadotropin concentrations, which suggests impaired activity of the hypothalamic–pituitary–gonadal axis in daughters of untreated or inadequately treated women at the level of ovaries. Interestingly, there were differences between girls and boys in the pattern of sex hormone production, not only in case of descendants of healthy mothers but also if they were born to mothers with thyroid hypofunction uncontrolled or poorly controlled during pregnancy. Irrespectively of the study group, salivary estrogen concentrations were higher and its detection period was longer in infant girls than in infant boys, which contrasted with more pronounced changes in testosterone in boys [19]. This observation is in agreement with autoptic studies demonstrating larger content of estrogens in the ovaries than in the testes of children suddenly dying during the first two years of life [30]. Differences in the detection periods and the lack of correlations between salivary estradiol and androgen indicate that decreased estrogen concentrations in daughters of untreated or inadequately treated hypothyroid women result from impaired gonadal estrogen production and/or are secondary to increased activity of aromatase, but cannot be explained by altered androgen production. The association with impaired activation of gonadotropes is supported by positive correlations between salivary estradiol and urinary LH concentrations. However, LH concentrations were detectable in saliva for a shorter period of time than estradiol, and this observation may suggest either high sensitivity of ovarian follicles to this hormone (they may be activated by very low and undetectable LH levels), or the presence of additional activators of estradiol production. Moreover, the presented explanation is in line with observations that in early pregnancy, thyroid hormones (which during this period are almost exclusively of maternal origin) stimulate estradiol secretion by trophoblast cells and increase aromatase activity [31]. Thus, it cannot be excluded that the impact of low thyroid hormone status on estrogen production persists in the first months after birth.

Differences in activation of the hypothalamic–pituitary–gonadal axis were paralleled by differences in the breast diameter and uterine length, which were greater in children born to either healthy mothers or to women with compensated thyroid function than in daughters of hypothyroid women who were untreated or undertreated during pregnancy. Moreover, both diameters positively correlated with estradiol concentrations, while in the offspring of women with normal gestational thyroid function there were also positive correlations between the breast size and FSH concentrations. Thus, minipuberty is associated with biological effects in the reproductive system, which may be less expressed if maternal hypothyroidism is during pregnancy poorly controlled. Interestingly, we observed an early reduction in uterine length and breast diameter, attributed to a withdrawal of placental estrogens in the first days of life, but less expressed than in the study by Kuiri-Hänninen et al. [16]. This difference can be explained by a later beginning of our study compared to theirs, which was associated with a relatively long-term interval between cessation of the exposition to maternal estrogens and study commencement. Interestingly, between-group differences in the breast diameter and uterine length were statistically significant only from month 5. This finding indicates that biological effects of estrogens require long-term stimulation of estrogen receptors. Moreover, it may suggest that the hormonal milieu related to minipuberty plays a greater role in the development of target organs than the hormonal milieu associated with pregnancy. It is worth noting that, at the end of the study, both diameters were still smaller in the descendants of untreated or inadequately substituted hypothyroid women than in the remaining girls. The clinical relevance of this finding remains unknown and requires further research. Undoubtedly, it would be reasonable to specify whether this difference persists to the late prepubertal period and is implicated in the final (pubertal) stage of physical maturation.

Another interesting observation resulting from our study is that changes in activity of the hypothalamic–pituitary–ovarian axis and in the size of target organs seem to be a direct consequence of the low thyroid hormone status. Two findings allow us to draw this conclusion. Firstly, female descendants of women who had been effectively supplemented (adequate levothyroxine doses) during gestation were characterized by an unaltered course of minipuberty. Secondly, there were no difference between both groups of hypothyroid women (untreated/inadequately substituted and adequately substituted) in the relative prevalence of disorders leading to thyroid hypofunction. Thus, our findings indicate that potentially unfavorable hormonal and clinical changes associated with minipuberty in female descendants of women with impaired thyroid secretory function may be prevented if hypothyroidism is effectively controlled over the entire period of pregnancy.

Lastly, the obtained results provide evidence that the altered course of female minipuberty is not related to thyroid autoimmunity itself. This observation is in disagreement with the impact of autoimmunity on the pubertal phase of development. Thyroid hypofunction diagnosed in the prepubertal age, which, in this population, is usually secondary to autoimmune thyroid disease [32], may delay puberty [33] or result in incomplete isosexual precocity in girls (development of breast and internal genitalia without appearance of pubic hair) [34]. Moreover, other autoimmune disorders developing before puberty are often complicated by delayed puberty, including juvenile idiopathic arthritis [35], inflammatory bowel diseases [36], celiac disease [37], and type 1 diabetes [38]. Moreover, the presence of autoimmunity in children was found to be accompanied by slow progression of puberty, isolated delayed menarche in females, and decreased duration and intensity of puberty growth [35]. Two findings contradict the association between impaired hypothalamic–pituitary–ovarian axis activity in the early childhood and autoimmune thyroid disease. Firstly, there were no differences in hormone concentrations between infant girls born to mothers with thyroid hypofunction of autoimmune and non-autoimmune origin. Secondly, there were also no correlations between concentrations of gonadotropins and steroids and titers of thyroid antibodies. Thus, thyroid peroxidase and thyroglobulin antibodies, despite the ability to penetrate the placental barrier [39], do not seem to have an impact the course of minipuberty in the offspring.

Some study limitations deserve to be noted. Although the study was sufficiently powered for group comparisons that were statistically significant, the relatively small sample size limits the extent to which our findings can be generalized. Because of its design (a small cohort study), we cannot exclude selection and confounding bias, which might have affected the obtained results. Relatively late recruitment of patients (the end of the first month of life) has not allowed us to investigate the earliest phase of minipuberty (the first few days after birth). Steroid hormone immunoassays used in the current study may theoretically interfere with compounds with structural similarities to the target steroid of the assay [40], possibly elevating salivary hormone levels. Because the Polish population is iodine-sufficient and selenium-deficient, the effect of gestational hypothyroidism does not have to be the same in populations with inadequate iodine [41] and sufficient selenium [42] status. Strict study inclusion and exclusion criteria make it impossible to draw conclusions concerning the course of minipuberty in children born to hypothyroid mothers with comorbidities and/or chronically treated during pregnancy with other drugs. Lastly, taking numerous precautions during study design and data analysis limited, but did not completely eliminate, the regression toward the mean [43].

## 4. Materials and Methods

This research was a single-center, prospective, matched, outpatient cohort study, carried out between May 2022 and April 2024. Owing to its nature, clinical trial registration was not required. The study design was reviewed and approved by the local bioethical commission (the Bioethical Committee of the Medical University of Silesia [approval number: PCN/CBN/0052/KB1/17/22, 12 April 2022), and the study was executed in compliance with the Declaration of Helsinki. Written informed consent was obtained from the parents of all infants participating in the study. Methodology and other study details adhere to the Strengthening the Reporting of Observational Studies in Epidemiology guidelines for reporting observational studies.

### 4.1. Participants

The research included a group of 96 female infants supervised by the principal study investigator (Karolina Kowalcze). The participants were chosen from a larger sample of children meeting the inclusion and exclusion criteria (n = 175) with the aim of matching the groups for women’s age, BMI, and gestational age of delivery. The matching algorithm has been described in our previous study [19]. Based on maternal thyroid status during pregnancy, the study population was divided into three groups. Groups 1 and 2 encompassed female descendants of women with hypothyroidism. Thirty-two daughters of women who, during pregnancy, had been untreated or insufficiently treated were enrolled into group 1. In this group, circulating thyrotropin concentrations in mothers on two or more gestational measurements during pregnancy (at least six weeks apart) exceeded 4.0 mU/L. Thirty-two girls born to mothers adequately supplemented with levothyroxine were assigned into group 2. Adequate supplementation was defined as all thyrotropin concentrations (three or more) during gestation in the reference range for pregnant women established by the American Thyroid Association [26] (between the lower limit of normal reduced by 0.4 mU/L and 4.0 mU/L in the first trimester, or between 0.5 and 4.0 mU/L in the remaining trimesters). Group 3 included 32 female descendants of healthy women. To be included into this group, the following criteria were required to be met: (a) thyrotropin concentrations on at least two measurements within the last 12 months (including at least one assessment in the first trimester of gestation) had to be between the lower limit of normal reduced in the first trimester of pregnancy by 0.4 mU/L and 4.0 mU/L; (b) free thyroxine, free triiodothyronine, and thyroid antibodies (thyroid peroxidase and thyroglobulin antibodies), if measured, had to be within normal limits; (c) the mother presented no signs and symptoms of thyroid dysfunction prior to or during gestation; and (d) the mothers had normal thyroid size and structure on ultrasound performed during pregnancy or at the first study visit (if thyroid ultrasound was not performed during pregnancy). The power analysis showed that 26 patients per group were needed to detect 25% between-group differences in gonadotropin and estradiol levels with a power of 80% and a two-tailed alpha level of 5%. Thus, considering the number of participants initiating and completed the study, the study was adequately powered to identify meaningful results. 

The infants were excluded if (a) maternal thyrotropin concentration was elevated on only one occasion during gestation, (b) the woman was diagnosed with isolated hypothyroxinemia (free thyroxine levels below 10.2 pmol/L coexisting with thyrotropin concentrations within the reference range), or (c) hormonal results were inconclusive. We also excluded girls if their mothers (a) were diagnosed with other chronic diseases; (b) were admitted to hospital during gestation or postpartum owing to any acute complications; (c) during pregnancy or lactation, received any medication for more than 7 days (except for thyroid hormone preparations and/or vitamin/micronutrient supplements for pregnant and lactating women), or (d) were alcohol- or drug-addicted. The remaining exclusion criteria included the following: birth before gestational age of 36 weeks, a history of birth asphyxia, congenital infections, major congenital anomalies, metabolic diseases, genetic syndromes, other chronic disorders, and any chronic pharmacotherapy.

### 4.2. Study Design

The infants were followed-up once a month for the first six months of life, once every two months for the following six months, and once every three months until the age of eighteen months (11 visits). At each visit, the parents were asked to assess the child’s health status and medication usage. Moreover, the investigator thoroughly examined the infant and interpreted the results of laboratory and imaging tests (if done). Saliva and urine samples were collected only if the girl was considered healthy and, except for mandatory vaccination, did not receive any medicine for at least 10 days prior to the visit. The results were statistically analyzed only if the infant attended at least 8 of all scheduled visits.

All anthropometric measurements were carried out using standard procedures by the principle investigator. Recumbent length was measured from the top of the head to the sole of the feet with 1 mm precision using a mobile infantometer (Seca, Hamburg, Germany). Head circumference was measured using a flexible measuring tape as the maximum circumference between the supraorbital ridge and the occipital prominence. Weight was determined using a digital infant scale (Seca 834, Hamburg, Germany) with 10 g precision after removing clothing and diapers. Weight-for-length in infants was converted to percentiles using the World Health Organization growth charts. BMI (in mothers) was calculated as weight in kilograms divided by squared length in meters.

Uterine length and ovarian volume were measured by the principle investigator using a high-resolution transducer 5-MHz to 12-MHz (Esaote MyLab Six, Genoa, Italy). Three independent measurements were always taken, and the obtained results were averaged. Uterine length was measured in the long axis from the top of the fundus to the distal aspects of the cervix (external os). The volume of each ovary was calculated using the following formula: 0.52 × length × depth × width. The first two dimensions were determined on parasaggital and the third on transverse images. If both ovaries could be visualized and measured in all three dimensions, the final volume was the average value for both ovaries. Otherwise, the size of the only visible ovary (right or left) was used as the ovarian volume. The number of follicles was also recorded. The mammary gland was assessed according to the method described by Henriksen et al. [44]. After identifying, by palpation, breast tissue as a firm subcutaneous disc, its diameter was measured to the nearest 1 mm using a caliper (Insize Europe, Zamudio, Spain). Diameters less than 3 mm were regarded as unmeasurable (as equivalent to the diameter of the nipple), and their size was assumed at 1 mm. Both glands were measured, and the mean diameter was the average of these two measurements.

### 4.3. Laboratory Assays

Urine and saliva samples were obtained between 7.00 and 8.30 a.m. Urine samples were collected using collection bags purchased from Medicavera (Szczecin, Poland). After cleaning and wiping the privates, the ends of the bag were attached on the skin above the privates and in the back of the vagina, while the sides next to each leg. Saliva samples were collected by the principal investigator by aspirating the saliva from the floor of the mouth with a 2 mL syringe. The procedure was performed at least one hour after the last feeding in order to prevent contamination and to limit a possible effect of feeding on hormone secretion [19,20]. Saliva collection, lasting for 25–50 s, was well tolerated and did not generate stress for the participants. The collected urine and saliva samples were then kept at −20 °C until analyzed.

All assays were carried out by a person who was unaware of the patient’s assignment. To increase the reliability of the results, the measurements were conducted in duplicate, and the obtained results were averaged. Urine FSH and LH levels were determined using a solid-phase, two-site chemiluminescent immunometric assay (Siemens Healthcare Diagnostics, Erlangen, Germany). Gonadotropin concentrations were then corrected for creatinine to standardize for the concentration of urine collected. Urine creatinine was measured using a modified Jaffe method (Roche Diagnostics, Basel, Switzerland). Concentrations of estradiol, testosterone, androstenedione, DHEA-S, progesterone, and 17-hydroxyprogesterone in saliva were measured by an enzyme-linked immuno-sorbent assay using reagents obtained from BioVendor R&D (Brno, Czech Republic), Diametra (Perugia, Italy), and IBL International (Hamburg, Germany). Limits of detection (LOD) were as follows: 4 pmol/L (estradiol), 10 pmol/L (testosterone), 18 pmol/L (androstenedione), 100 nmol/L (DHEA-S), 16 pmol/L (progesterone), 11 pmol/L (17-hydroxyprogesterone), 0.1 U/L (FSH), and 0.1 U/L (LH). The same methodology was used as in our previous studies [19,20]. 

### 4.4. Statistical Analysis

All variables were log-transformed to ensure that they closely followed a normal distribution. Within-patient differences at different time points were compared by repeated measures analysis of variance, followed by a post hoc analysis using Tukey’s Honestly Significantly Different test. Comparisons between the groups were performed by repeated measures analysis of variance, with post hoc analysis carried out using Bonferroni’s test. Associations between categorical variables were determined using the chi-square test. Correlations were evaluated using Pearson’s r tests (between two continuous variables), phi coefficient (between one continuous and one categorical variable), and point-biserial (between two categorical variables). The standardized mean difference (SMD) or the standardized difference of proportions (for percentage data) were used to calculate the effect size. In all analyses, differences were regarded as statistically significant if two-tailed *p* values were below 0.05 and/or 95% confidence intervals did not include the null value.

## 5. Conclusions

The obtained results indicate that thyroid hypofunction during gestation, if not substituted or adequately substituted, seems to have an impact on the course of minipuberty in their daughters. Female descendants of such women are characterized by shorter and less pronounced activation of the hypothalamic–pituitary–ovarian axis in early childhood and by less evident changes in the target organs (ovaries, uterus, and breast) than in daughters of mothers with normal thyroid function. Thus, the obtained results suggest that maternal hypothyroidism, irrespectively of its clinical and biochemical severity, requires early detection and accurate substitution during pregnancy. Due to numerous study limitations, further research is warranted to confirm our findings and to understand better the molecular mechanisms underlying the obtained results. Lastly, it seems interesting to investigate a relationship between other maternal thyroid disorders during pregnancy and minipuberty.

## Figures and Tables

**Table 1:** Baseline characteristics of the study population.

Variable	Group 1	Group 2	Group 3	SMD * (95% Confidence Interval)		
				1 vs. 2	1 vs. 3	2 vs. 3
Number (n)	29	29	30	-	-	-
Gestational age of delivery (weeks)	39 ± 2	40 ± 2	40 ± 2	−0.493 (−1.021, 0.025)	−0.493 (−1.016, 0.020)	0 (−0.510, −0.510)
Birth order: first/second/third and subsequent (%)	45/45/14	38/45/17	47/50/13	0.108 (−0.203, 0.418)	0.046 (−0.122, 0.214)	0.132 (−0.242, 0.506)
Body length (cm)	53.8 ± 1.7	53.5 ± 1.6	54.2 ± 1.6	0.179 (−0.335, 0.697)	−0.239 (−0.754, 0.271)	−0.432 (−0.952, 0.081)
Body weight (kg)	4.35 ± 0.49	4.29 ± 0.50	4.37 ± 0.53	0.120 (−0.394, 0.636)	−0.039 (−0.549, 0.471)	−0.153 (−0.666, 0.357)
Weight-for-length percentile	68 ± 23	63 ± 20	59 ± 22	0.229 (−0.286, 0.747)	0.395 (−0.117, 0.914)	0.188 (−0.322, 0.701)
Head circumference (cm)	36.9 ± 0.6	36.7 ± 0.8	36.6 ± 0.7	0.279 (−0.236, 0.799)	0.453 (−0.069, 0.975)	0.131 (−0.378, 0.644)
Thyrotropin concentration (mU/L)	7.4 ± 2.7	7.1 ± 2.5	7.0 ± 2.9	0.114 (−0.400, 0.630)	0.141 (−0.369, 0.653)	0.036 (−0.474, 0.547)
Breastfeeding (%)	83	79	80	0.215 (−0.290, 0.720)	0.196 (−0.206, 0.598)	−0.055 (−0.238, 0.128)

The data are shown as the mean ± standard deviation (unless otherwise stated); thyrotropin concentration was assessed in dried whole blood spots, collected from neonates between days 3 and 5 after birth. * For percentage data, the standardized difference of proportions was calculated instead of SMD. Group 1: daughters born to women with hypothyroidism untreated or inadequately substituted during gestation; Group 2: daughters born to hypothyroid women treated during gestation with adequate doses of levothyroxine; Group 3: daughters of healthy women. Abbreviation: SMD—standardized mean difference.

**Table 2:** Baseline characteristics of mothers of the infant girls participating in the study.

Variable	Group 1	Group 2	Group 3	SMD * (95% Confidence Interval)		
				1 vs. 2	1 vs. 3	2 vs. 3
Number (n)	29	29	30	-	-	-
Age (years)	34 ± 8	32 ± 8	32 ± 9	0.247 (−0.268, 0.766)	0.231 (−0.278, 0.746)	0 (−0.510, 0.510)
University/secondary/primary or vocational education (%)	36/39/24	41/34/24	40/37/23	−0.197 (−0.692, 0.298)	−0.135 (−0.526, 0.257)	0.067 (−0.374, 0.508)
Employment rate/white-collar/pink-collar/blue-collar workers (%)	83/28/28/28	79/28/28/24	80/30/30/20	0.056 (−0.403, 0.516)	0.119 (−0.348, 0.586)	−0.126 (−0.469, 0.218)
Smokers (%)	28	24	27	0.102 (−0.206, 0.410)	0.068 (−0.295, 0.432)	−0.167 (−0.580, 0.246)
BMI (kg/m^2^)	24.8 ± 3.9	24.3 ± 3.2	24.2 ± 3.5	0.138 (−0.375, 0.655)	0.160 (−0.350, 0.673)	0.029 (−0.481, 0.540)
Systolic blood pressure (mmHg)	121 ± 17	119 ± 18	117 ± 26	0.113 (−0.401, 0.629)	0.179 (−0.331, 0.692)	0.088 (−0.422, 0.599)
Diastolic blood pressure (mmHg)	80 ± 7	79 ± 7	78 ± 6	0.141 (−0.374, 0.658)	0.303 (−0.208, 0.819)	0.152 (−0.358, 0.664)

The data are shown as the mean ± standard deviation (unless otherwise stated); * for percentage data, the standardized difference of proportions was calculated instead of SMD. Group 1: women with hypothyroidism untreated or inadequately substituted during gestation; Group 2: hypothyroid women treated during gestation with adequate doses of levothyroxine; Group 3: healthy women. Abbreviations: BMI—body mass index; SMD—standardized mean difference.

**Table 3:** Salivary estradiol levels in female infants participating in the study.

Age	Group 1	Group 2	Group 3	SMD (95% Confidence Interval)		
				1 vs. 2	1 vs. 3	2 vs. 3
Month 1	22 ± 10	39 ± 16	38 ± 14	−1.257 (−1.835, −0.703)	−1.294 (−1.870, −0.743)	0.065 (−0.444, 0.577)
Month 2	24 ± 12	34 ± 11	40 ± 13	−0.644 (−1.180, −0.122)	−1.261 (−1.834, −0.712)	−0.491 (−1.014, 0.023)
Month 3	20 ± 14	40 ± 22	42 ± 28	−1.070 (−1.633, −0.528)	−0.976 (−1.526, −0.443)	−0.078 (−0.589, 0.432)
Month 4	21 ± 10	35 ± 12	37 ± 15	−1.250 (−1.828, −0.697)	−1.234 (−1.806, −0.687)	−0.145 (−0.657, 0.365)
Month 5	15 ± 8 ^a^	31 ± 15	30 ± 18	−1.313 (−1.897, −0.756)	−1.056 (−1.613, −0.520)	0.060 (−0.450, 0.570)
Month 6	10 ± 6 ^b^	25 ± 12 ^a^	22 ± 10 ^b^	−1.560 (−2.167, −0.983)	−1.430 (−2.019, −0.869)	0.268 (−0.242, 0.784)
Month 8	Below LOD ^c^	22 ± 8 ^b^	20 ± 12 ^b^	−3.045 (−3.849, −2.312)	−1.316 (−1.894, −0.763)	0.193 (−0.317, 0.706)
Month 10	Below LOD ^c^	15 ± 8 ^c^	14 ± 7 ^c^	−1.064 (−1.626, −0.523)	−1.009 (−1.562, −0.475)	0.131 (−0.378, 0.644)

The data are expressed in pmol/L and shown as the mean ± standard deviation. From month 12 to month 18, estradiol levels were below LOD in all groups. For between-group comparisons, LOD value was assigned for estradiol in group 1 at months 8 and 10. Group 1: daughters born to women with hypothyroidism untreated or inadequately substituted during gestation; Group 2: daughters born to hypothyroid women treated during gestation with adequate doses of levothyroxine; Group 3: daughters of healthy women. ^a^ *p* < 0.05 vs. levels during the first 4 months of life in the same study group; ^b^ *p* < 0.05 vs. levels during the first 5 months of life in the same study group; ^c^ *p* < 0.05 vs. levels during the first 6 months of life in the same study group; Abbreviations: LOD—limit of detection; SMD—standardized mean difference.

**Table 4:** Salivary testosterone concentrations in female infants participating in the study.

Age	Group 1	Group 2	Group 3	SMD (95% Confidence Interval)		
				1 vs. 2	1 vs. 3	2 vs. 3
Month 1	50 ± 23	47 ± 22	52 ± 25	0.131 (−0.383, 0.648)	−0.082 (−0.593, 0.428)	−0.209 (−0.723, 0.301)
Month 2	58 ± 31	51 ± 24	56 ± 20	0.249 (−0.265, 0.768)	0.076 (−0.434, 0.587)	−0.228 (−0.738, 0.286)
Month 3	49 ± 18	46 ± 19	51 ± 22	0.159 (−0.354, 0.677)	−0.098 (−0.607, 0.412)	−0.240 (−0.754, 0.270)
Month 4	53 ± 21	44 ± 20	46 ± 24	0.433 (−0.084, 0.958)	0.305 (−0.205, 0.822)	−0.089 (−0.601, 0.421)
Month 5	47 ± 20	39 ± 15	40 ± 18	0.446 (−0.071, 0.972)	0.363 (−0.148, 0.881)	−0.059 (−0.570, 0.450)

The data are expressed in pmol/L and shown as the mean ± standard deviation. In all groups, testosterone concentrations were below LOD from month 6 to month 18. Group 1: daughters born to women with hypothyroidism untreated or inadequately substituted during gestation; Group 2: daughters born to hypothyroid women treated during gestation with adequate doses of levothyroxine; Group 3: daughters of healthy women. Abbreviation: SMD—standardized mean difference.

**Table 5:** Salivary androstenedione concentrations in female infants participating in the study.

Age	Group 1	Group 2	Group 3	SMD (95% Confidence Interval)		
				1 vs. 2	1 vs. 3	2 vs. 3
Month 1	75 ± 30	69 ± 35	80 ± 41	0.182 (−0.332, 0.699)	−0.134 (−0.649, 0.373)	−0.284 (−0.800, 0.226)
Month 2	82 ± 41	78 ± 36	76 ± 34	0.102 (−0.412, 0.618)	0.157 (−0.352, 0.670)	0.056 (−0.454, 0.567)
Month 3	90 ± 47	83 ± 42	81 ± 43	0.155 (−0.359, 0.672)	0.197 (−0.313, 0.711)	0.046 (−0.464, 0.557)
Month 4	84 ± 39	80 ± 36	74 ± 35	0.105 (−0.409, 0.621)	0.267 (−0.244, 0.782)	0.167 (−0.342, 0.680)
Month 5	78 ± 40	76 ± 38	70 ± 32	0.050 (−0.464, 0.566)	0.218 (−0.292, 0.732)	0.169 (−0.341, 0.682)

The data are expressed in pmol/L and shown as the mean ± standard deviation. In all groups, androstenedione concentrations were below LOD from month 6 to month 18. Group 1: daughters born to women with hypothyroidism untreated or inadequately substituted during gestation; Group 2: daughters born to hypothyroid women treated during gestation with adequate doses of levothyroxine; Group 3: daughters of healthy women. Abbreviation: SMD—standardized mean difference.

**Table 6:** Salivary DHEA-S concentrations in female infants participating in the study.

Age	Group 1	Group 2	Group 3	SMD (95% Confidence Interval)		
				1 vs. 2	1 vs. 3	2 vs. 3
Month 1	182 ± 79	156 ± 81	173 ± 61	0.321 (−0.195, 0.842)	0.126 (−0.384, 0.638)	−0.234 (−0.749, 0.276)
Month 2	162 ± 84	170 ± 71	181 ± 87	−0.101 (−0.617, 0.413)	−0.219 (−0.733, 0.291)	−0.136 (−0.649, 0.373)
Month 3	142 ± 73	155 ± 61	164 ± 68	−0.191 (−0.708, 0.323)	−0.328 (−0.824, 0.203)	−0.137 (−0.650, 0.372)
Month 4	156 ± 50	168 ± 43	159 ± 58	−0.254 (−0.773, 0.261)	−0.055 (−0.566, 0.455)	0.173 (−0.336, 0.686)
Month 5	139 ± 58	178 ± 55	155 ± 57	−0.681 (−1.678, 0.003)	−0.275 (−0.790, 0.236)	0.405 (−0.107, 0.925)
Month 6	152 ± 71	180 ± 86	163 ± 71	−0.350 (−0.872, 0.165)	−0.153 (−0.665, 0.357)	0.213 (−0.297, 0.727)
Month 8	173 ± 62	167 ± 75	152 ± 76	0.086 (−0.428, 0.602)	0.298 (−0.212, 0.814)	0.196 (−0.314, 0.709)
Month 10	160 ± 82	155 ± 60	149 ± 65	0.069 (−0.446, 0.584)	0.147 (−0.363, 0.659)	0.095 (−0.415, 0.606)
Month 12	140 ± 60	148 ± 62	146 ± 51	−0.129 (−0.646, 0.385)	−0.106 (−0.618, 0.403)	0.035 (−0.475, 0.546)

The data are expressed in nmol/L and shown as the mean ± standard deviation. In all groups, DHEA-S concentrations were below LOD at months 15 and 18. Group 1: daughters born to women with hypothyroidism untreated or inadequately substituted during gestation; Group 2: daughters born to hypothyroid women treated during gestation with adequate doses of levothyroxine; Group 3: daughters of healthy women. Abbreviations: DHEA-S—dehydroepiandrosterone sulfate; SMD—standardized mean difference.

**Table 7:** Salivary progesterone concentrations in female infants participating in the study.

Age	Group 1	Group 2	Group 3	SMD (95% Confidence Interval)		
				1 vs. 2	1 vs. 3	2 vs. 3
Month 1	75 ± 45	70 ± 46	68 ± 40	0.108 (−0.406, 0.624)	0.162 (−0.347, 0.675)	0.046 (−0.464, 0.557)
Month 2	65 ± 42	59 ± 32	55 ± 34	0.158 (−0.355, 0.675)	0.259 (−0.252, 0.774)	0.119 (−0.390, 0.631)
Month 3	70 ± 41	62 ± 37	65 ± 32	0.202 (−0.312, 0.720)	0.134 (−0.375, 0.647)	−0.086 (−0.597, 0.424)
Month 4	82 ± 47	71 ± 43	74 ± 35	0.240 (−0.273, 0.760)	0.191 (−0.319, 0.704)	−0.076 (−0.587, 0.434)
Month 5	68 ± 34	76 ± 46	59 ± 41	−0.195 (−0.713, 0.319)	0.235 (−0.275, 0.750)	0.385 (−0.126, 0.904)
Month 6	64 ± 30	69 ± 36	60 ± 34	−0.149 (−0.665, 0.365)	0.123 (−0.387, 0.635)	0.254 (−0.256, 0.767)
Month 8	60 ± 40	64 ± 37	55 ± 29	−0.102 (−0.618, 0.412)	0.142 (−0.368, 0.653)	0.268 (−0.243, 0.783)
Month 10	58 ± 35	55 ± 29	50 ± 26	0.092 (−0.422, 0.608)	0.257 (−0.254, 0.772)	0.179 (−0.331, 0.692)
Month 12	55 ± 38	52 ± 24	49 ± 28	0.093 (−0.421, 0.609)	0.178 (−0.332, 0.691)	0.113 (−0.396, 0.625)

The data are expressed in pmol/L and shown as the mean ± standard deviation. In all groups, progesterone concentrations were LOD at months 15 and 18. Group 1: daughters born to women with hypothyroidism untreated or inadequately substituted during gestation; Group 2: daughters born to hypothyroid women treated during gestation with adequate doses of levothyroxine; Group 3: daughters of healthy women. Abbreviation: SMD—standardized mean difference.

**Table 8:** Salivary 17-hydroxyprogesterone concentrations in female infants participating in the study.

Age	Group 1	Group 2	Group 3	SMD (95% Confidence Interval)		
				1 vs. 2	1 vs. 3	2 vs. 3
Month 1	58 ± 32	63 ± 41	65 ± 43	−0.134 (−0.651, 0.380)	−0.182 (−0.695, 0.328)	−0.046 (−0.558, 0.463)
Month 2	61 ± 34	60 ± 38	59 ± 37	0.027 (−0.487, 0.542)	0.055 (−0.454, 0.566)	0.026 (−0.484, 0.537)
Month 3	53 ± 24	58 ± 40	55 ± 35	−0.126 (−0.643, 0.388)	−0.054 (−0.565, 0.456)	0.078 (−0.431, 0.590)
Month 4	59 ± 28	61 ± 39	53 ± 28	−0.058 (−0.573, 0.456)	0.211 (−0.299, 0.725)	0.233 (−0.277, 0.747)
Month 5	48 ± 31	60 ± 41	53 ± 38	−0.325 (−0.847, 0.189)	−0.142 (−0.654, 0.368)	0.175 (−0.335, 0.688)
Month 6	43 ± 28	55 ± 32	48 ± 35	−0.394 (−0.917, 0.123)	−0.155 (−0.668, 0.354)	0.206 (−0.304, 0.719)
Month 8	46 ± 26	58 ± 31	50 ± 31	−0.414 (−0.938, 0.103)	−0.138 (−0.650, 0.372)	0.255 (−0.256, 0.770)
Month 10	50 ± 30	48 ± 26	52 ± 28	0.070 (−0.444, 0.586)	−0.068 (−0.579, 0.442)	−0.204 (−0.718, 0.306)
Month 12	47 ± 23	46 ± 30	49 ± 30	0.037 (−0.477, 0.552)	−0.074 (−0.585, 0.436)	−0.099 (−0.610, 0.411)

The data are expressed in pmol/L and shown as the mean ± standard deviation. In all groups, 17-hydroxyprogesterone concentrations were below LOD at months 15 and 18. Group 1: daughters born to women with hypothyroidism untreated or inadequately substituted during gestation; Group 2: daughters born to hypothyroid women treated during gestation with adequate doses of levothyroxine; Group 3: daughters of healthy women. Abbreviation: SMD—standardized mean difference.

**Table 9:** Urinary FSH levels in female infants participating in the study.

Age	Group 1	Group 2	Group 3	SMD (95% Confidence Interval)		
				1 vs. 2	1 vs. 3	2 vs. 3
Month 1	0.80 ± 0.46	1.55 ± 0.62	1.72 ± 0.84	−1.355 (−1.943, −0.795)	−1.334 (−1.915,−0.780)	−0.227 (−0.741, 0.283)
Month 2	0.85 ± 0.50	1.68 ± 0.78	1.78 ± 0.92	−1.250 (−1.828, −0.697)	−1.234 (−1.804, −0.686)	−0.115 (−0.627, 0.394)
Month 3	0.78 ± 0.53	1.75 ± 0.83	1.69 ± 0.88	−1.374 (−1.963, −0.812)	−1.231 (−1.802, −0.683)	0.069 (−0.441, 0.580)
Month 4	0.69 ± 0.42	1.53 ± 0.79	1.62 ± 0.82	−1.310 (−1.893, −0.753)	−1.401 (−1.988, −0.842)	−0.110 (−0.622, 0.399)
Month 5	0.73 ± 0.34	1.60 ± 0.88	1.56 ± 0.74	−1.286 (−1.868, −0.731)	−1.414 (−2.002, −0.854)	0.049 (−0.461, 0.559)
Month 6	0.78 ± 0.39	1.72 ± 0.90	1.64 ± 0.68	−1.337 (−1.923, −0.778)	−1.524 (−2.123, −0.956)	0.099 (−0.411, 0.611)
Month 8	0.70 ± 0.38	1.49 ± 0.78	1.70 ± 0.85	−1.270 (−1.850, −0.716)	−1.490 (−2.085, −0.924)	−0.254 (−0.769, 0.256)
Month 10	0.40 ± 0.29 ^a^	1.55 ± 0.80	1.46 ± 0.80	−1.732 (−2.358, −1.141)	−1.727 (−2.,347, −1.142)	0.111 (−0.399, 0.623)
Month 12	Below LOD ^a^	0.95 ± 0.54 ^b^	0.87 ± 0.52 ^b^	−2.187 (−2.868, −1.553)	−2.039 (−2.696, −1.425)	0.149 (−0.361, 0.661)

The data are expressed in international units per mmol of creatinine and shown as the mean ± standard deviation. At months 15 and 18, FSH levels were below LOD in all groups. For between-group comparisons, LOD value was assigned for FSH in group 1 at month 12. Group 1: daughters born to women with hypothyroidism untreated or inadequately substituted during gestation; Group 2: daughters born to hypothyroid women treated during gestation with adequate doses of levothyroxine; Group 3: daughters of healthy women. ^a^ *p* < 0.05 vs. levels during the first 8 months of life in the same study group; ^b^ *p* < 0.05 vs. levels during the first 10 months of life in the same study group. Abbreviations: FSH—follicle-stimulating hormone; LOD—limit of detection; SMD—standardized mean difference.

**Table 10:** Urinary LH levels in female infants participating in the study.

Age	Group 1	Group 2	Group 3	SMD (95% Confidence Interval)		
				1 vs. 2	1 vs. 3	2 vs. 3
Month 1	0.73 ± 0.48	1.12 ± 0.55	1.02 ± 0.49	−0.745 (−1.286, −0.219)	−0.590 (−1.117, −0.073)	0.190 (−0.320, 0.703)
Month 2	0.69 ± 0.46	1.18 ± 0.58	1.20 ± 0.63	−0.923 (−1.475, −0.389)	−0.910 (−1.456, −0.380)	−0.0343 (−0.543, 0.478)
Month 3	0.65 ± 0.39	1.25 ± 0.60	1.22 ± 0.65	−1.170 (−1.741, −0.622)	−1.045 (−1.601, −0.509)	0.047 (−0.463, 0.558)
Month 4	0.59 ± 0.31	1.05 ± 0.53	1.14 ± 0.58	−1.045 (−1.606, −0.505)	−1.162 (−1.727, −0.618)	−0.177 (−0.690, 0.332)
Month 5	0.55 ± 0.28	0.98 ± 0.43	1.01 ± 0.53	−1.169 (−1.740, −0.621)	−1.066 (−1.623, −0.528)	−0.061 (−0.572, 0.449)
Month 6	Below LOD ^a^	0.64 ± 0.32 ^a^	0.70 ± 0.30 ^a^	−2.326 (−3.026, −1.678)	−2.731 (−3.480, −2.041)	−0.191 (−0.704, 0.319)
Month 8	Below LOD ^a^	0.40 ± 0.22 ^b^	0.43 ± 0.25 ^b^	−1.855 (−2.496, −1.254)	−1.792 (−2.419, −1.200)	−0.126 (−0.638, 0.384)

The data are expressed in international units per mmol of creatinine and shown as the mean ± standard deviation. From month 10 to month 18, LH levels were below LOD in all groups. For between-group comparisons, LOD value was assigned for LH in group 1 at months 6 and 8. Group 1: daughters born to women with hypothyroidism untreated or inadequately substituted during gestation; Group 2: daughters born to hypothyroid women treated during gestation with adequate doses of levothyroxine; Group 3: daughters of healthy women. ^a^ *p* < 0.05 vs. levels during the first 5 months of life in the same study group; ^b^ *p* < 0.05 vs. levels during the first 6 months of life in the same study group. Abbreviations: LH—luteinizing hormone; LOD—limit of detection; SMD—standardized mean difference.

**Table 11:** Ovarian volume in female infants participating in the study.

Age	Group 1	Group 2	Group 3	SMD (95% Confidence Interval)		
				1 vs. 2	1 vs. 3	2 vs. 3
Month 1	0.80 ± 0.18	0.76 ± 0.22	0.80 ± 0.30	0.196 (−0.318, 0.714)	0 (−0.510, 0.510)	−0.150 (−0.662, 0.360)
Month 2	0.82 ± 0.23	0.98 ± 0.25 ^a^	0.96 ± 0.27 ^a^	−0.657 (−1.192, −0.134)	−0.550 (−1.076, −0.035)	0.076 (−0.434, 0.587)
Month 3	0.79 ± 0.26	1.01 ± 0.30 ^a^	1.06 ± 0.42 ^a^	−0.773 (−1.315, −0.246)	−0.760 (−1.297, −0.237)	−0.135 (−0.647, 0.375)
Month 4	0.81 ± 0.20	0.97 ± 0.24 ^a^	1.02 ± 0.38 ^a^	−0.714 (−1.253, −0.189)	−0.937 (−1.485, −0.406)	−0.206 (−0.719, 0.304)
Month 5	0.76 ± 0.30	1.00 ± 0.34 ^a^	0.97 ± 0.28 ^a^	−0.738 (−1.278, −0.212)	−0.715 (−1.249, −0.194)	0.095 (−0.415, 0.607)
Month 6	0.82 ± 0.21	0.95 ± 0.25 ^a^	1.06 ± 0.40 ^a^	−0.555 (−1.086, −0.035)	−0.737 (−1.273, −0.216)	−0.324 (−0.841, 0.187)
Month 8	0.61 ± 0.20 ^b^	1.03 ± 0.40 ^a^	0.95 ± 0.35 ^a^	−1.310 (−1.894, −0.753)	−1.172 (−1.738, −0.628)	0.210 (0.300, 0.724)
Month 10	0.60 ± 0.32 ^b^	0.95 ± 0.30 ^a^	0.94 ± 0.28 ^a^	−1.096 (−1.661, −0.552)	−1.117 (−1.679, −0.577)	0.034 (−0.477, 0.544)
Month 12	0.62 ± 0.26 ^b^	0.80 ± 0.25 ^c^	0.78 ± 0.30 ^c^	−0.696 (−1.234, −0.172)	−0.562 (−1.088, −0.046)	−0.071 (−0.583, 0.439)
Month 15	0.59 ± 0.21 ^b^	0.76 ± 0.23 ^c^	0.80 ± 0.32 ^c^	−0.762 (−1.303, −0.235)	−0.763 (−1.300, −0.240)	−0.141 (−0.654, 0.369)
Month 18	0.56 ± 0.28 ^b^	0.74 ± 0.26 ^c^	0.78 ± 0.24 ^c^	−0.657 (−1.193, −0.134)	−0.834 (−1.375, −0.308)	−0.158 (−0.671, 0.352)

The data are expressed in mL and shown as the mean ± standard deviation. Group 1: daughters born to women with hypothyroidism untreated or inadequately substituted during gestation; Group 2: daughters born to hypothyroid women treated during gestation with adequate doses of levothyroxine; Group 3: daughters of healthy women. ^a^ *p* < 0.05 vs. value at month 1 in the same study group; ^b^ *p* < 0.05 vs. values during the first 6 months of life in the same study group; ^c^ *p* < 0.05 vs. values during the first 10 months of life in the same study group. Abbreviation: SMD—standardized mean difference.

**Table 12:** Uterine length in female infants participating in the study.

Age	Group 1	Group 2	Group 3	SMD (95% Confidence Interval)		
				1 vs. 2	1 vs. 3	2 vs. 3
Month 1	32 ± 5	34 ± 5	34 ± 4	−0.395 (−0.918, 0.122)	−0.437 (−0.957, 0.076)	0 (−0.510, 0.510)
Month 2	28 ± 4 ^a^	31 ± 5 ^a^	30 ± 5 ^a^	−0.506 (−1.034, 0.012)	−0.435 (−0.956, 0.078)	0.197 (−0.313, 0.711)
Month 3	28 ± 5 ^a^	30 ± 5 ^a^	29 ± 4 ^a^	−0.395 (−0.918, 0.122)	−0.218 (−0.732, 0.292)	0.218 (−0.292, 0.732)
Month 4	27 ± 4 ^a^	29 ± 4 ^a^	29 ± 5 ^a^	−0.493 (−1.021, 0.025)	−0.435 (−0.956, 0.078)	0 (−0.511, 0.510)
Month 5	24 ± 3 ^c^	30 ± 4 ^a^	29 ± 4 ^a^	−2.790 (−3.555, −2.088)	−1.392 (−1.978, −0.834)	0.247 (−0.263, 0.761)
Month 6	25 ± 4 ^c^	29 ± 3 ^a^	30 ± 4 ^a^	−1.116 (−1.683, −0.571)	−1.234 (−1.805, −0.686)	−0.278 (−0.794, 0.232)
Month 8	24 ± 4 ^c^	29 ± 4 ^a^	29 ± 3 ^a^	−1.234 (−1.809, −0.681)	−1.398 (−1.985, −0.840)	0 (−0.510, 0.510)
Month 10	25 ± 3 ^c^	29 ± 4 ^a^	30 ± 5 ^a^	−1.116 (−1.682, −0.571)	−1.192 (−1.759, −0.647)	−0.217 (−0.731, 0.292)
Month 12	26 ± 4 ^b^	30 ± 3 ^a^	30 ± 4 ^a^	−1.116 (−1.682, −0.571)	−0.986 (−1.538, −0.454)	0 (−0.510, 0.510)
Month 15	25 ± 4 ^c^	30 ± 4 ^a^	29 ± 4 ^a^	−1.233 (−1.809, −0.681)	−0.987 (−1.538, −0.454)	0.247 (−0.263, 0.761)
Month 18	25 ± 3 ^c^	30 ± 4 ^a^	29 ± 5 ^a^	−1.395 (−1.986, −0.832)	−0.953 (−1.502, −0.422)	0.217 (−0.292, 0.731)

The data are expressed in mm and shown as the mean ± standard deviation. Group 1: daughters born to women with hypothyroidism untreated or inadequately substituted during gestation; Group 2: daughters born to hypothyroid women treated during gestation with adequate doses of levothyroxine; Group 3: daughters of healthy women. ^a^ *p* < 0.05 vs. value at month 1 in the same study group; ^b^ *p* < 0.05 vs. values during the first 3 months of life in the same study group; ^c^ *p* < 0.05 vs. values during the first 4 months of life in the same study group. Abbreviation: SMD—standardized mean difference.

**Table 13:** Breast diameter in female infants participating in the study.

Age	Group 1	Group 2	Group 3	SMD (95% Confidence Interval)		
				1 vs. 2	1 vs. 3	2 vs. 3
Month 1	11 ± 4	13 ± 5	13 ± 4	−0.436 (−0.961, 0.081)	−0.493 (−1.016, 0.021)	0 (−0.510, 0.510)
Month 2	7 ± 4 ^a^	9 ± 4 ^a^	10 ± 5 ^a^	−0.493 (−1.037, 0.041)	−0.653 (−1.318, 0.001)	−0.218 (−0.731, 0.293)
Month 3	7 ± 3 ^a^	9 ± 4 ^a^	10 ± 5 ^a^	−0.532 (−1.081, 0.008)	−0.692 (−1.382, 0.002)	−0.218 (−0.731, 0.293)
Month 4	6 ± 4 ^a^	8 ± 4 ^a^	8 ± 5 ^a^	−0.493 (−1.021, 0.025)	−0.435 (−0.956, 0.078)	0 (−0.510, 0.510)
Month 5	5 ± 2 ^a^	8 ± 4 ^a^	8 ± 5 ^a^	−0.936 (−1.489, −0.401)	−0.773 (−1.310, −0.250)	0 (−0.510, 0.510)
Month 6	5 ± 3 ^a^	9 ± 4 ^a^	8 ± 4 ^a^	−1.116 (−1.682, −0.571)	−0.835 (−1.376, −0.310)	0.247 (−0.264, 0.761)
Month 8	6 ± 3 ^a^	9 ± 4 ^a^	9 ± 4 ^a^	−0.837 (−1.383, −0.307)	−0.835 (−1.376, −0.310)	0 (−0.510, 0.510)
Month 10	6 ± 2 ^a^	9 ± 3 ^a^	8 ± 4 ^a^	−1.161 (−1.731, −0.613)	−0.621 (−1.150, −0.103)	0.278 (−0.232, 0.794)
Month 12	7 ± 3 ^a^	10 ± 4 ^a^	10 ± 5 ^a^	−0.837 (−1.383, −0.307)	−0.717 (−1.250, −0.194)	0 (−0.510, 0.510)
Month 15	6 ± 4 ^a^	10 ± 3 ^a^	9 ± 4 ^a^	−1.116 (−1.682, −0.571)	−0.740 (−1.280, −0.218)	0.278 (−0.232, 0.794)
Month 18	7 ± 4 ^a^	10 ± 4 ^a^	9 ± 3 ^a^	−0.740 (−1.280, −0.214)	−0.560 (−1.086, −0.044)	0.280 (−0.231, 0.795)

The data are expressed in mm and shown as the mean ± standard deviation. Group 1: daughters born to women with hypothyroidism untreated or inadequately substituted during gestation; Group 2: daughters born to hypothyroid women treated during gestation with adequate doses of levothyroxine; Group 3: daughters of healthy women. ^a^ *p* < 0.05 vs. value at month 1 in the same study group. Abbreviation: SMD—standardized mean difference.

**Table 14:** Estradiol and gonadotropin concentrations infant daughters born to women with hypothyroidism untreated or poorly controlled during pregnancy.

Age	Autoimmune Hypothyroidism	Hypothyroidism of Non-Autoimmune Origin	SMD (95% Confidence Interval)
Estradiol			
Month 1	95 ± 30	105 ± 39	−0.281 (−1.019, 0.446)
Month 2	108 ± 25	91 ± 34	0.557 (−0.176, 1.312)
Month 3	102 ± 34	97 ± 30	0.151 (−0.575, 0.884)
Month 4	94 ± 37	106 ± 29^,^	−0.350 (−1.091, 0.378)
Month 5	110 ± 43	89 ± 30	0.547 (−0.186, 1.301)
Month 6	106 ± 40	93 ± 29	0.360 (0.368, 1.102)
FSH			
Month 1	100 ± 31	101 ± 34	−0.030 (−0.759, 0.698)
Month 2	104 ± 36	95 ± 40	0.230 (−0.496, 0.966)
Month 3	108 ± 40	91 ± 35	0.439 (−0.291, 1.185)
Month 4	97 ± 30	104 ± 32	−0.220 (−0.955, 0.507)
Month 5	102 ± 29	98 ± 31	0.130 (−0.597, 0.861)
Month 6	98 ± 28	103 ± 31	−0.165 (0.898, 0.562)
Month 8	103 ± 34	96 ± 20	0.242 (−0.485, 0.978)
Month 10	95 ± 38	106 ± 28	−0.319 (−1.058, 0.409)
LH			
Month 1	90 ± 32	111 ± 43	−0.541 (−1.295, 0.191)
Month 2	98 ± 28	102 ± 28	−0.139 (−0.871, 0.588)
Month 3	103 ± 35	96 ± 31	0.205 (−0.521, 0.940)
Month 4	105 ± 30	94 ± 22	0.404 (−0.325, 1.149)
Month 5	95 ± 29	106 ± 31	−0.357 (−1.098, 0.371)

The data are shown as the percentage of the mean value in the whole group 1 ± standard deviation. Comparisons were only made at time points at which hormone levels in group 1 were above LOD Abbreviations: FSH—follicle-stimulating hormone; LH—luteinizing hormone; LOD—limit of detection; SMD—standardized mean difference.

**Table 15:** Comparisons between the course of minipuberty in girls and boys born to mothers with gestational hypothyroidism.

Parameter	Infant Boys	Infant Girls
Urinary FSH	lower concentrations than in sons of healthy women; no difference in detection period	lower concentrations and shorter detection period than in daughters of healthy women; similar concentrations but longer detection period than in sons of women with gestational hypothyroidism
Urinary LH	lower concentrations than in sons of healthy women; no difference in detection period	lower concentrations and shorter detection period than in daughters of healthy women; slightly lower concentrations and shorter detection period than in sons of women with gestational hypothyroidism
Salivary testosterone	lower concentrations and shorter detection period than in sons of healthy women	no differences in concentrations and detection period compared with daughters of healthy women; lower concentrations but similar detection period compared with sons of women with gestational hypothyroidism
Salivary androstenedione	similar concentrations but shorter detection period than in sons of healthy women	no differences in concentrations and detection period compared with daughters of healthy women and sons of women with gestational hypothyroidism
Salivary estradiol	no differences in concentrations and detection period compared with sons of healthy women	lower concentrations and shorter detection period than in daughters of healthy women; higher concentrations and longer detection period than in sons of women with gestational hypothyroidism
Salivary DHEA-S, progesterone and 17-hydroxyprogesterone	no differences in concentrations and detection periods compared with sons of healthy women	no differences in concentrations and detection periods compared with daughters of healthy women; no differences in detection periods but slightly lower concentrations of progesterone and 17-hydroxyprogesterone (but not DHEA-S) compared with sons of women with gestational hypothyroidism
Genital organs	testicular volume and penile length smaller than in sons of healthy women	ovarian volume, uterine length, and breast size smaller than in daughters of healthy women
Association with thyroid autoimmunity	thyroid autoimmunity does not explain differences in hormone concentrations and in sizes of genital organs	thyroid autoimmunity does not explain differences in hormone concentrations and in sizes of genital organs
Impact of adequate levothyroxine substitution during pregnancy	no differences in hormone concentrations and sizes of genital organs between sons of women with hypothyroidism adequately controlled during pregnancy and sons of healthy women	no differences in hormone concentrations and sizes of genital organs between daughters of women with hypothyroidism adequately controlled during pregnancy and daughters of healthy women

Data concerning infant boys are based on our previous research [19]. Unless otherwise stated, data refer to infants of mothers with hypothyroidism uncontrolled or poorly controlled during pregnancy. Abbreviations: DHEA-S—dehydroepiandrosterone sulfate; FSH—follicle-stimulating hormone; LH—luteinizing hormone.

## Data Availability

The data that support the findings of this study are available from the corresponding author upon reasonable request.

## Abbreviations

DHEA-S—dehydroepiandrosterone sulfate; dotp—depending on time point; FSH—follicle-stimulating hormone; LH—luteinizing hormone; LOD—limit of detection; SMD—standardized mean difference.
